# Selenium suppresses inflammation by inducing microRNA-146a in *Staphylococcus aureus*-infected mouse mastitis model

**DOI:** 10.18632/oncotarget.20740

**Published:** 2017-09-08

**Authors:** Weijing Sun, Qi Wang, Yingfang Guo, Yifan Zhao, Xinying Wang, Zhenbiao Zhang, Ganzhen Deng, Mengyao Guo

**Affiliations:** ^1^ College of Veterinary Medicine, Huazhong Agricultural University, Wuhan 430070, People’s Republic of China

**Keywords:** selenium, mastitis, S. aureus, miRNA-146, inflammation

## Abstract

We studied the effects of selenium (Se) on the inflammatory response in *Staphylococcus aureus* (*S. aureus*)-infected mastitis-model mice and mammary epithelial cells. In infected mice, Se elicited a dose-dependent decrease in mammary gland pathology that included inflammatory cell infiltration, disorganized acinar structure and mammary cell necrosis. Se decreased inflammation by increasing miR-146a and decreasing TLR2/6 as well as NF-κB and MAPK signaling pathways in mammary tissue from infected mice and mammary epithelial cells. A miR-146a inhibitor suppressed the anti-inflammatory effects of Se in infected mammary epithelial cells. Se, miR-146a and TLR2 were associated in determining the inflammatory response in mouse with infection-induced mastitis. Thus, Se inhibits pro-inflammatory responses in mammary tissues from *S. aureus*-infected mice by inducing miR-146a.

## INTRODUCTION

Selenium (Se) is an essential micronutrient that is beneficial for cardiovascular health, cancer prevention and optimal immune response [1, 2]. It has little effect on inflammation initiation, but effectively regulates the progression of inflammation [3]. Selenium also regulates progression of hepatitis, metritis and other infectious diseases [4]. Hence, the role of selenium in human disease is an area of active research.

Mastitis is an infectious disease of the mammary system in humans and animals [5]. *Staphylococcus aureus* (*S. aureus*) is the most common pathogen in viral infected mastitis [6]. Selenium regulates immunity against *S. aureus* mastitis [4]. The toll-like receptor 2 (TLR2) is the main receptor that recognizes *S. aureus* [7]. In response to exogenous *S. aureus*, TLR2 activates the downstream inflammatory signaling pathways leading to the release of cytokines, chemokines and interferons [8, 9]. MAPK and NF-κB signaling pathways co-ordinate and activate transcription factors, which enter the nucleus and transcribe various cytokines and chemokines [10]. Therefore, TLRs, NF-κB and MAPK signaling pathways are critical for inflammatory response.

MicroRNAs (miRNAs) are a class of non-coding single-stranded RNA molecules of about 22 nucleotides in length [11]. They regulate post-transcriptional gene expressions and therefore affect numerous critical cellular functions like metabolism, proliferation, cell survival and others. Many studies have demonstrated that some miRNAs alter inflammatory response by affecting TLR expression [12, 13]. MiR-146a regulates TLR2, TLR3 and TLR4 expression [14] and modulates the inflammatory response in otitis media and chronic periodontitis [15]. Moreover, selenium regulates the expression of multiple miRNAs such as miR-374, miR-16, miR-199a-5p, miR-155 and miR-30e [16]. However, the role of selenium in the expression of miR-146a and TLR immunity is not known. Therefore, in this study, we investigated the role of selenium in the regulation of miR-146a and its effects on *S. aureus* related mastitis.

## RESULTS

### Se alleviates mammary pathology in *S. aureus* infected mastitis model mice

**Figure 1:**
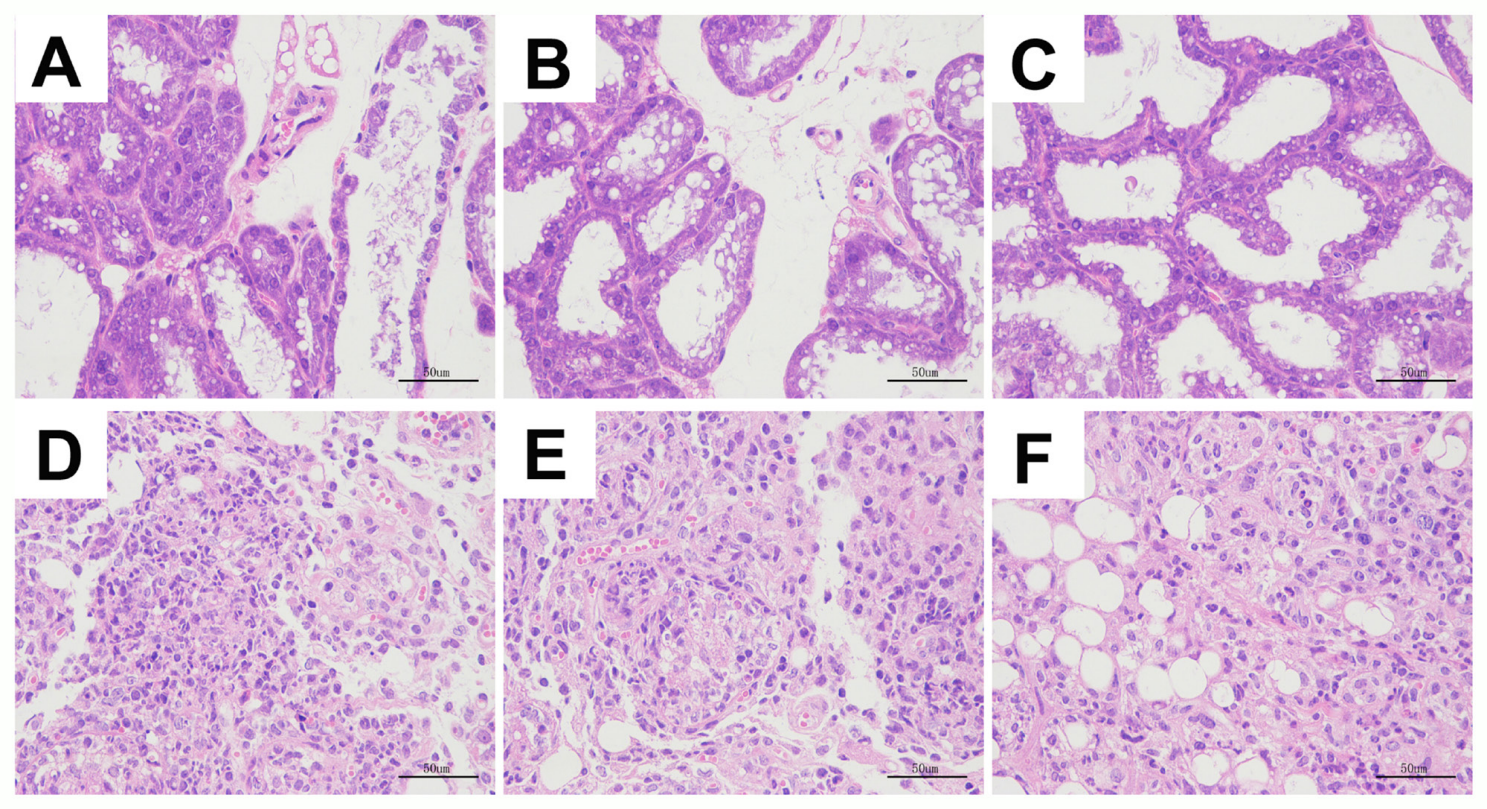
Se prevented the histopathological changes of mammary tissues from *S. aureus*-infected mastitis model mice Representative images (100X.) of H&E stained mammary tissue sections from **(A)** LG: uninfected mice on low (0.037 mg/kg) Se diet; **(B)** NG: uninfected mice on normal (0.15 mg/kg) Se diet; **(C)** HG: uninfected mice on high (1.5 mg/kg) Se diet; **(D)** SLG: *S. aureus* infected mice on low Se diet; **(E)** SNG: *S.aureus* infected mice on normal Se diet and **(F)** SHG: *S. aureus* infected mice on high Se diet.

We analyzed H&E stained mammary tissue sections from all groups of mice for histopathological changes. The mammary tissues from uninfected selenium treated mice (LG, NG, HG) showed normal cell morphology and histological structure (Figure 1). In the mammary tissues from *S. aureus* infected selenium treated mice (SLG, SNG, SHG), we observed pathological changes such as inflammatory cell infiltration, mammary epithelial cell shedding, disorganized acinar structure, mammary cell necrosis, nuclear concentration and nuclear dissolution (Figure 1). The histopathological changes were more drastic in the SLG (low Se concentration) than the SNG and SHG groups. This suggested that selenium alleviates mammary pathology in *S. aureus* infected mastitis model mice in a concentration dependent manner.

### Se increases cell survival in mammary tissues of *S. aureus*-infected mastitis model mice

**Figure 2:**
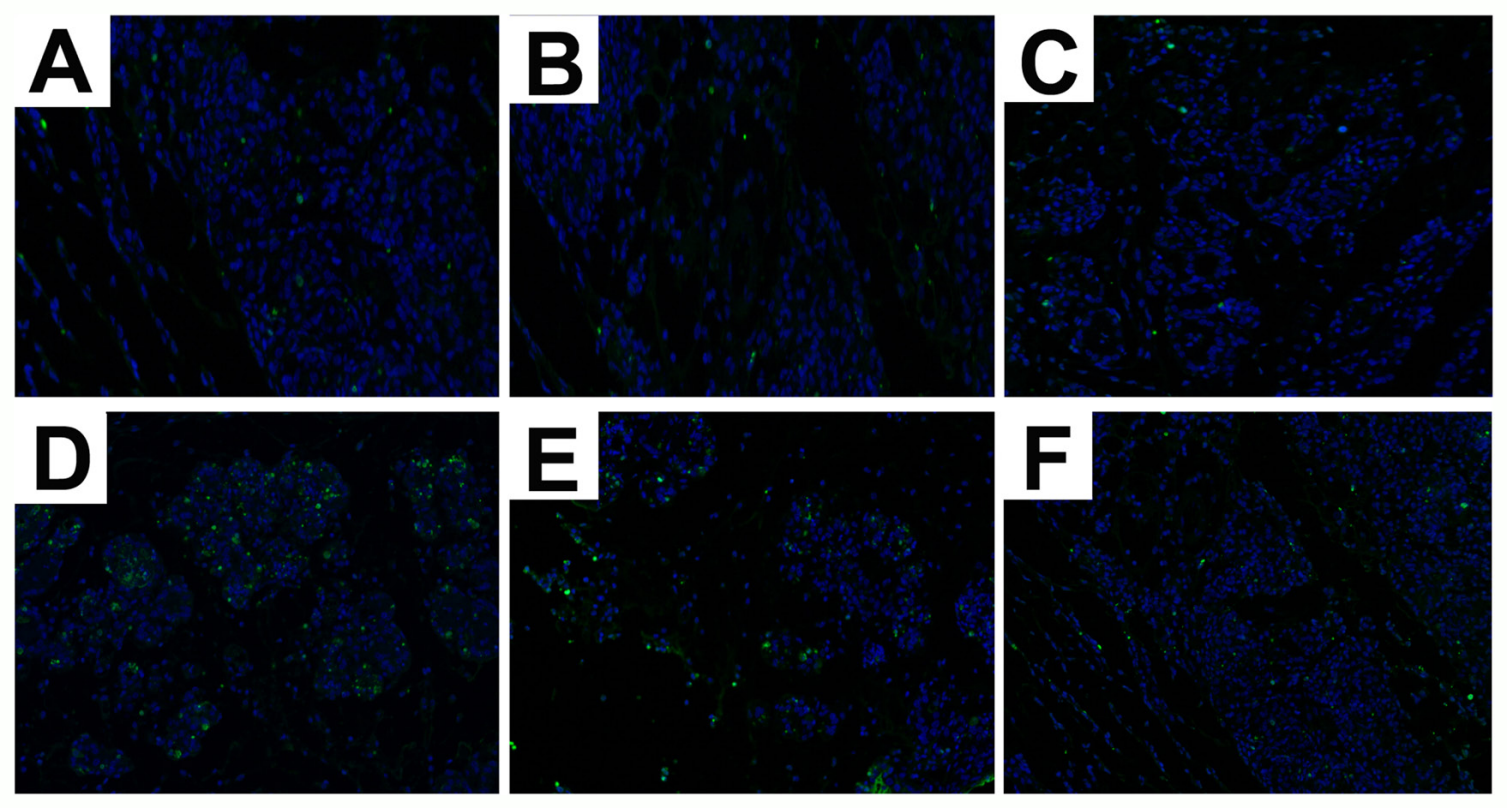
Effect of Se on apoptosis in mammary tissues from *S. aureus*-infected mastitis model mice Representative images of TUNEL stained mammary tissue sections from **(A)** LG: uninfected mice on low (0.037 mg/kg) Se diet; **(B)** NG: uninfected mice on normal (0.15 mg/kg) Se diet; **(C)** HG: uninfected mice on high (1.5 mg/kg) Se diet; **(D)** SLG: *S. aureus* infected mice on low Se diet; **(E)** SNG: *S.aureus* infected mice on normal Se diet and **(F)** SHG: *S. aureus* infected mice on high Se diet. Note: Green fluorescence indicates apoptotic cells.

TUNEL assay showed increased apoptosis in mammary tissues from *S. aureus*-infected selenium treated mice (SLG, SNG, SHG) than uninfected Se treated groups (LG, NG, HG). Moreover, SHG group demonstrated decreased apoptosis than SLG and SNG groups (Figure 2). This suggested that Se treatment increased mammary epithelial cell survival in *S. aureus*-infected mastitis model mice.

### Se decreases neutrophil activity in the mammary tissues from *S. aureus*-infected mastitis mice

**Figure 3:**
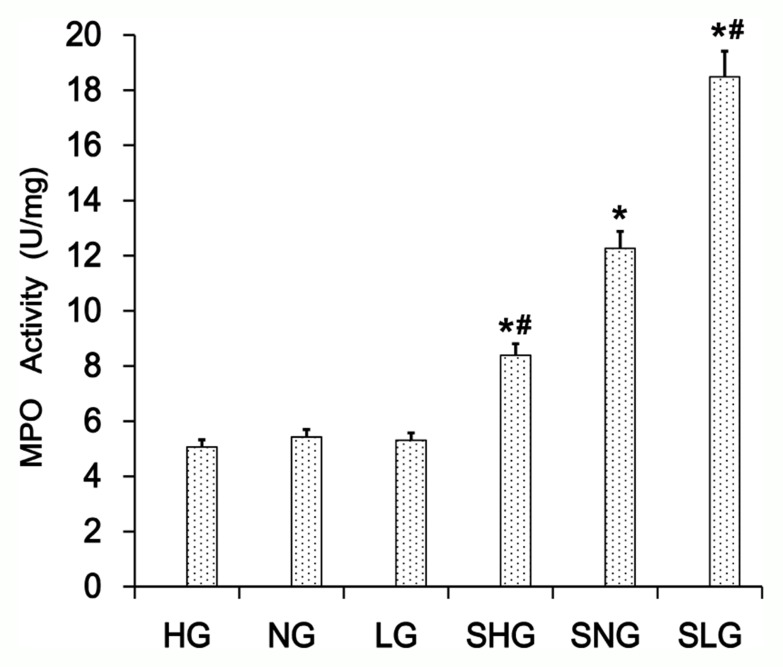
Effect of Se on MPO levels in mammary tissues from *S. aureus*-infected mastitis model mice MPO levels in mammary tissue extracts from. LG: uninfected mice on low (0.037 mg/kg) Se diet; NG: uninfected mice on normal (0.15 mg/kg) Se diet; HG: uninfected mice on high (1.5 mg/ kg) Se diet; SLG: *S. aureus* infected mice on low Se diet; SNG: S. aureus infected mice on normal Se diet and SHG: *S. aureus* infected mice on high Se diet. Note: ^*^ denotes P<0.05 compared to NG; ^#^ denotes P<0.05 compared to SNG.

Mammary tissues from *S. aureus*-infected with Se treated mice (SLG, SNG, SHG) showed increased MPO levels than uninfected Se treated groups (LG, NG, HG; Figure 3). MPO levels were highest in the SLG group (low Se) than in the SNG and SHG groups (Figure 3). This demonstrated that Se decreased neutrophil activity in the mammary tissues from *S. aureus*-infected mastitis mice in a concentration dependent manner.

### Se content in mammary tissues from mastitis model mice

**Figure 4:**
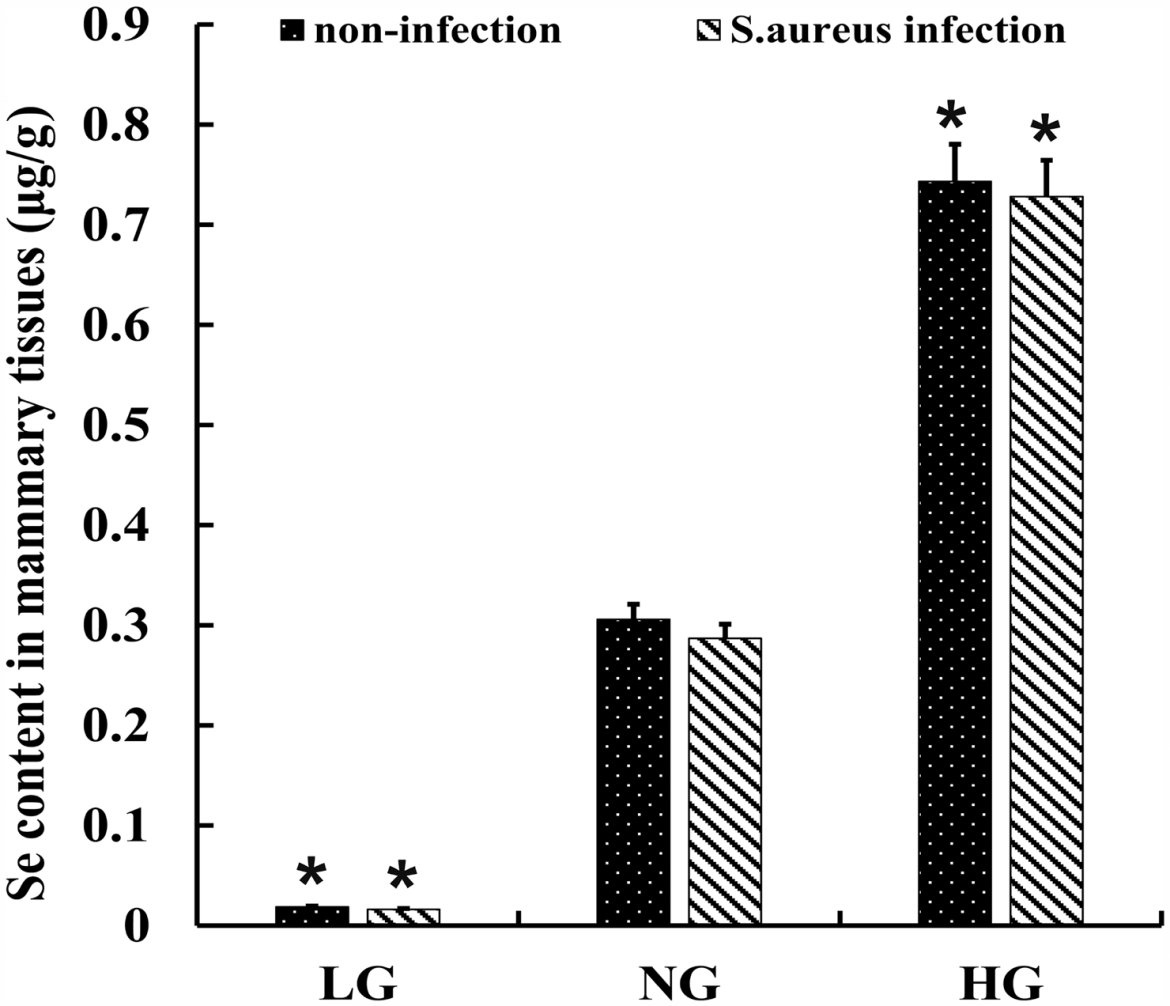
Se levels in mammary tissues from *S. aureus* infected mastitis model mice Selenium concentration in mammary tissues from. LG: uninfected mice on low (0.037 mg/kg) selenium diet; NG: uninfected mice on normal (0.15 mg/kg) selenium diet; HG: uninfected mice on high (1.5 mg/ kg) selenium diet; Note: ^*^ denotes P<0.05 compared to NG.

We assayed selenium levels in mammary tissues of the 6 groups of mastitis model mice and observed that it correlated with the dose of Se. Mammary tissues from HG and SHG groups showed the highest selenium levels (Figure 4). Moreover, the Se levels in HG and SHG groups were similar (Figure 4). Therefore, *S. aureus* infection did not alter its levels in the mammary tissues.

### Se decreases inflammatory response in mammary tissues in *S. aureus*-infected mastitis model mice

**Figure 5:**
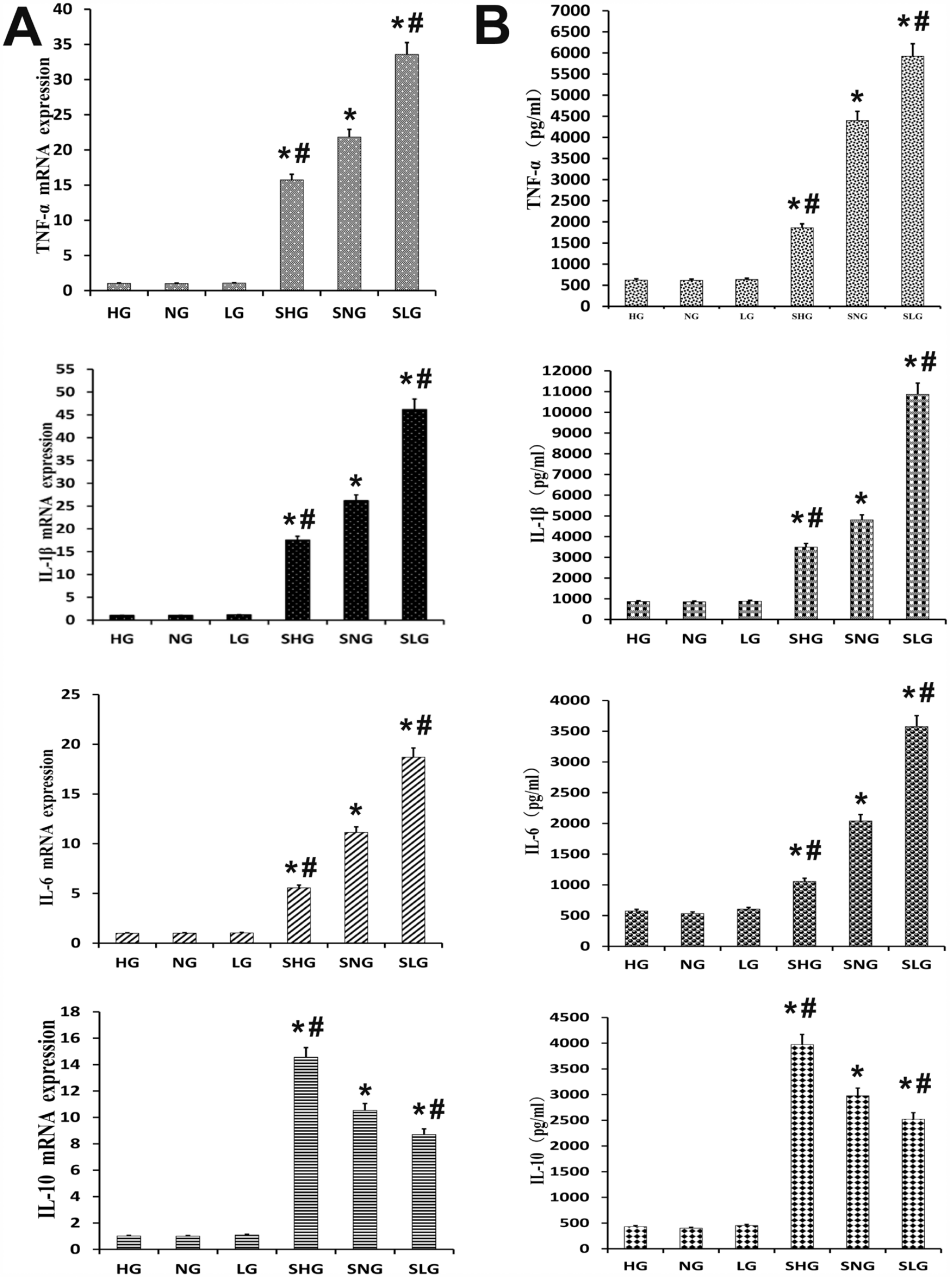
Effect of Se on inflammation in mammary tissues from *S. aureus* infected mastitis model mice **(A)** qRT-PCR analysis of TNF-α, IL-β, IL-6 and IL-10 mRNA expression in mammary tissues from mastitis model mice. **(B)** Estimation of TNF-α, IL-β, IL-6 and IL-10 protein levels by ELISA in mammary tissues from mastitis model mice. Note: LG: uninfected mice on low (0.037 mg/kg) Se diet; NG: uninfected mice on normal (0.15 mg/kg) Se diet; HG: uninfected mice on high (1.5 mg/kg) Se diet; SLG: *S. aureus* infected mice on low Se diet; SNG: *S.aureus* infected mice on normal Se diet and SHG: *S. aureus* infected mice on high Se diet. Data presented as the mean± SD (n = 10/group). ^*^ denotes P<0.05 compared to NG; ^#^denotes P<0.05 compared to SNG.

We analyzed the anti-inflammatory effects of Se on *S. aureus* mastitis by determining protein and mRNA levels of TNF-α, IL-1β, IL-6 and IL-10 by ELISA and qRT-PCR, respectively. The *S. aureus*-infected Se treated groups (SLG, SNG, SHG) showed higher TNF-α, IL-1β, IL-6 and IL-10 protein and mRNA levels than uninfected Se treated groups (LG, NG, HG). Moreover, TNF-α, IL-1β and IL-6 expression was highest in the SLG (low Se) group, whereas IL-10 levels were highest in the SHG (high Se) group (Figure 5A and 5B). This demonstrated that Se decreased inflammatory cytokine expression in *S. aureus*-infected mouse mammary tissues in a concentration dependent manner.

### Se decreases TLR2 and TLR6 levels in mammary tissues in *S. aureus* infected mastitis model mice

**Figure 6:**
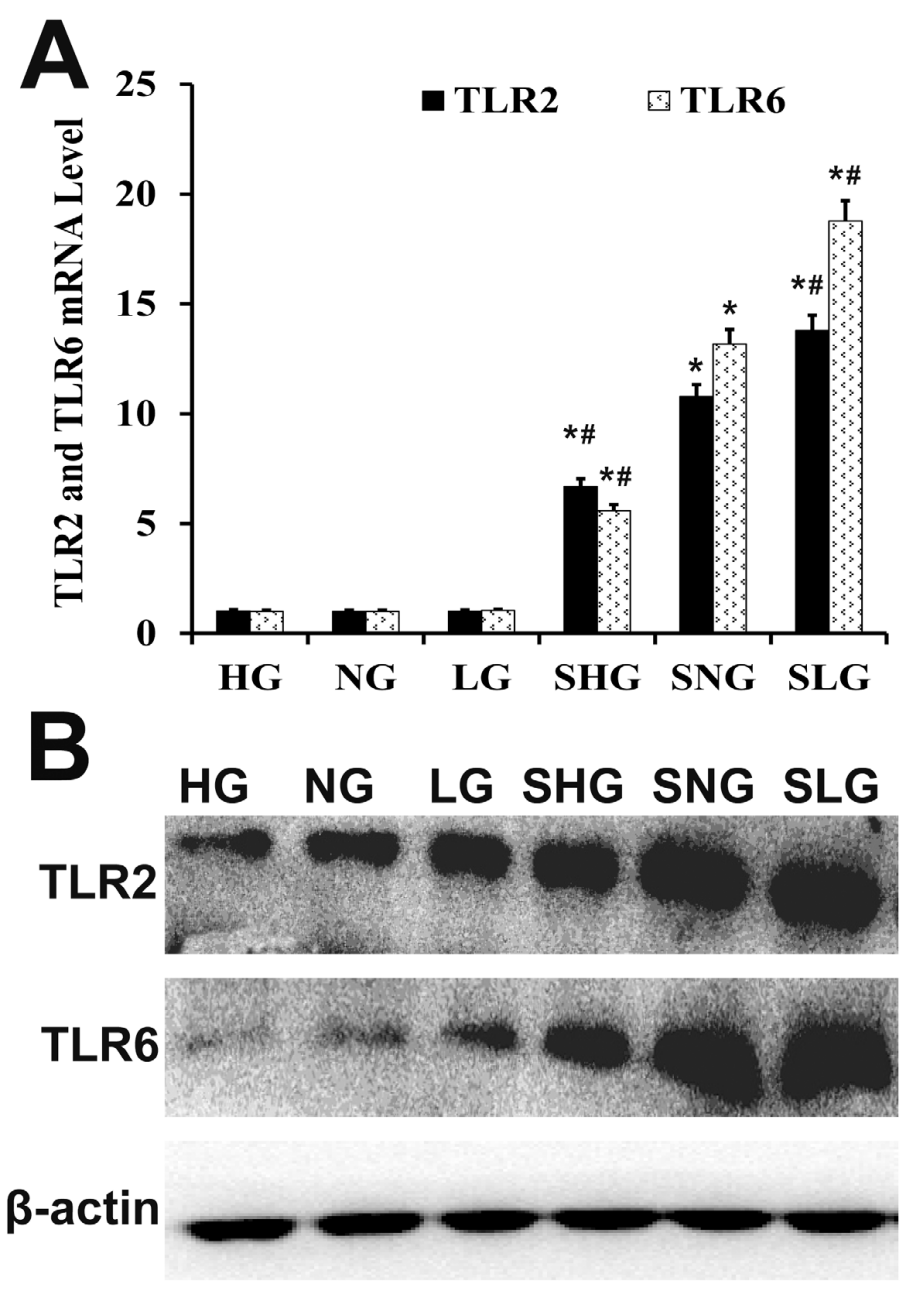
Effect of Se on TLR2 and TLR6 expression in mammary tissues from *S. aureus*-infected mastitis model mice **(A)** qRT-PCR and **(B)** western blot analysis of TLR2 and TLR6 mRNA and protein levels in mammary tissues from. LG: uninfected mice on low (0.037 mg/kg) Se diet; NG: uninfected mice on normal (0.15 mg/kg) Se diet; HG: uninfected mice on high (1.5 mg/ kg) Se diet; SLG: *S. aureus* infected mice on low Se diet; SNG: *S .aureus* infected mice on normal Se diet and SHG: *S. aureus* infected mice on high Se diet. Note: ^*^ denotes P<0.05 compared to NG; ^#^denotes P<0.05 compared to SNG.

TLR2 and TLR6 play a vital role in the inflammatory response against infections. We analyzed mRNA and protein levels of TLR2 and TLR6 by qRT-PCR and western blotting, respectively. -infected Se treated groups (SLG, SNG, SHG) showed higher TLR2 and TLR6 expression than uninfected Se treated groups (LG, NG, HG; Figure 6). TLR2 and TLR6 levels were highest in the SLG group than in the SNG and SHG groups (Figure 6).

### Se decreases NF-κB and MAPK signaling in mammary tissues in *S. aureus* infected mastitis model mice

**Figure 7:**
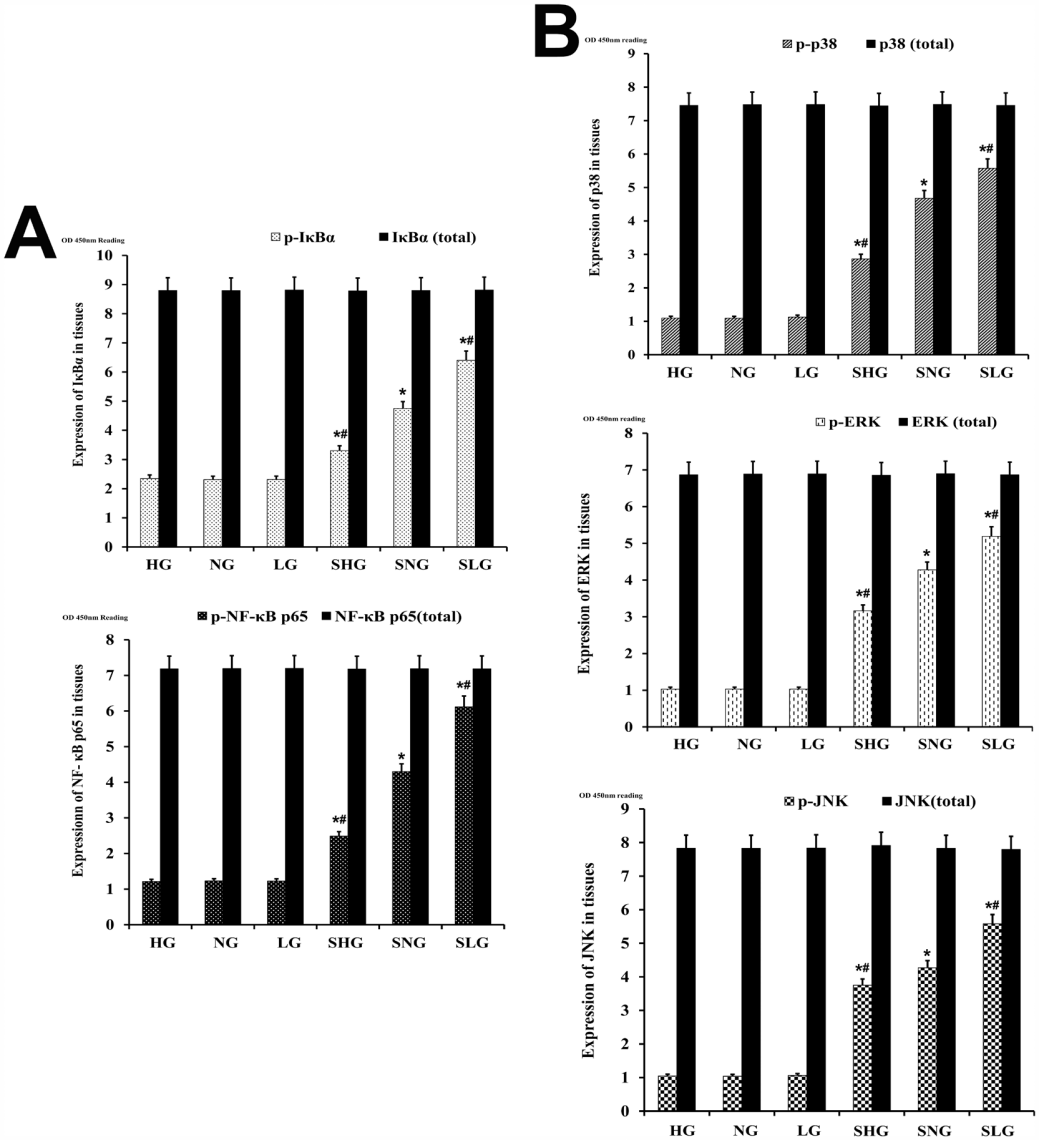
Effect of Se on NF-κB and MAPK signaling in mammary tissues from *S. aureus*-infected mastitis model mice Estimation of total and phosphorylated **(A)** NF-κB p65 and IκB-α as well as **(B)** p38, ERK and JNK protein levels in mammary tissues from mastitis model mice. Note: LG: uninfected mice on low (0.037 mg/kg) Se diet; NG: uninfected mice on normal (0.15 mg/kg) Se diet; HG: uninfected mice on high (1.5 mg/kg) Se diet; SLG: *S. aureus* infected mice on low Se diet; SNG: *S. aureus* infected mice on normal Se diet and SHG: *S. aureus* infected mice on high Se diet. ^*^ denotes P<0.05 compared to NG; ^#^denotes P<0.05 compared to SNG.

Phosphorylation of IκBα releases NF-κB p65 to translocate from the cytoplasm to the nucleus and transcribe genes encoding inflammatory cytokines [10]. NF-κB is complemented by co-ordinated activation of p38, JNK and ERK MAP kinases [8]. Therefore, we investigated the status of NF-κB and MAPK pathways in the mammary tissues from the different groups of mice with ELISA. *S. aureus*-infected Se treated groups (SLG, SNG, SHG) showed higher levels of phosphorylated p65, IκBα, p38, JNK and ERK proteins were than uninfected Se treated groups (LG, NG, HG; Figure 7). The non-phosphorylated p65, IκBα, p38, JNK and ERK proteins were similar in all groups. Moreover, phosphorylated forms of p65, IκBα, p38, JNK and ERK were higher in the SLG group than SNG and SHG groups (Figure 7). This suggested that Se treatment decreased NF-κB and MAPK signaling in a concentration dependent manner.

### Effect of Se on miRNA-146a Levels in mammary tissues of *S. aureus*-infected mastitis model mice

**Figure 8:**
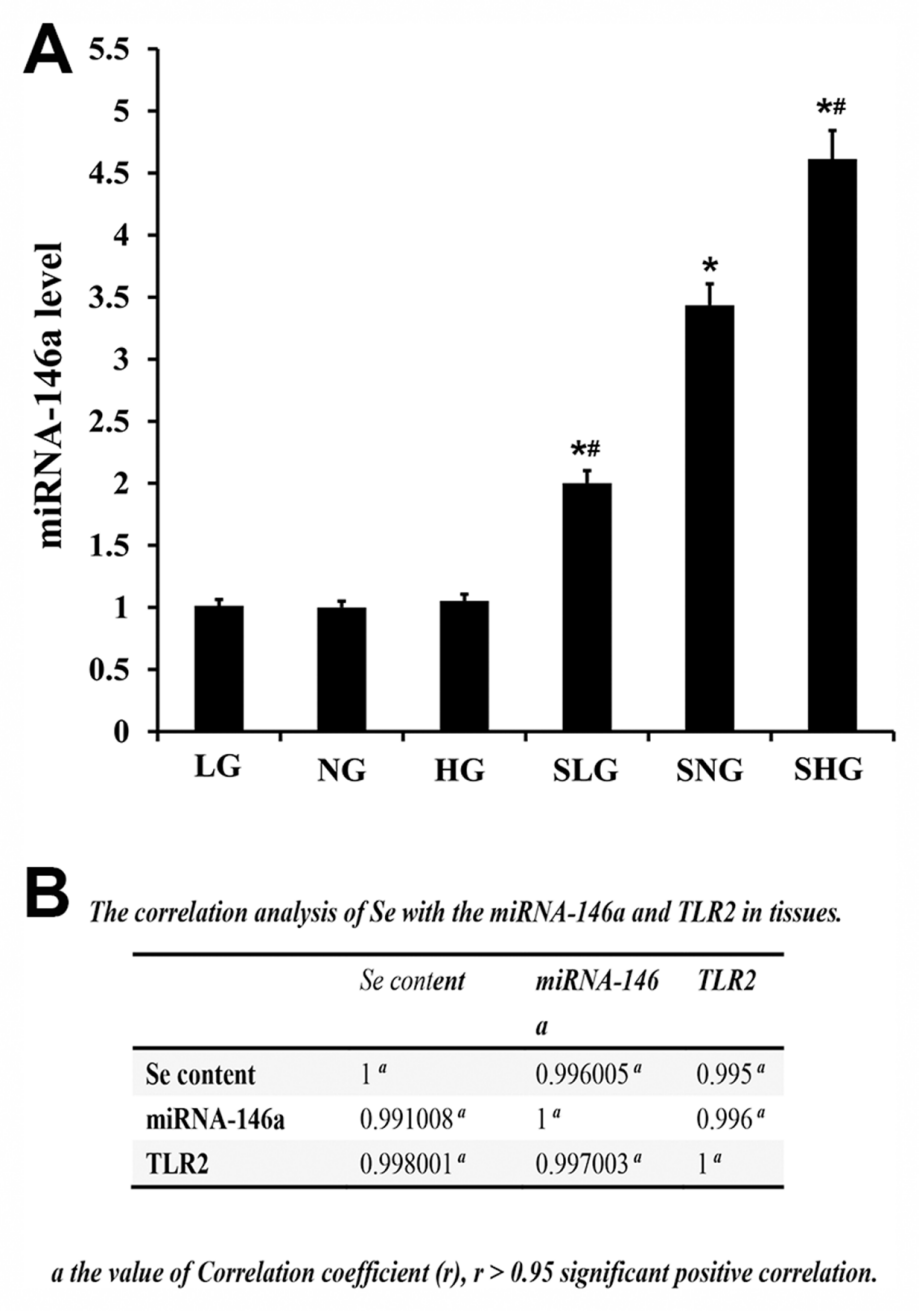
Effect of Se on miR-146a expression in mammary tissues from *S. aureus*- infected mastitis model mice qRT-PCR analysis of miR-146a levels in mammary tissues from mastitis model mice. Note: LG: uninfected mice on low (0.037 mg/kg) Se diet; NG: uninfected mice on normal (0.15 mg/kg) Se diet; HG: uninfected mice on high (1.5 mg/kg) Se diet; SLG: *S. aureus* infected mice on low Se diet; SNG: *S. aureus* infected mice on normal Se diet and SHG: *S. aureus* infected mice on high Se diet. ^*^ denotes P<0.05 compared to NG; ^#^denotes P<0.05 compared to SNG.

Next, we analyzed the effect of Se treatment on miR-146a levels by qRT-PCR. *S. aureus*-infected Se treated mice (SLG, SNG, SHG) showed higher miR146a levels than uninfected Se treated groups (LG, NG, HG; Figure 8). Moreover, miR-146a levels in the SHG group (high Se) were higher than SLG and SNG groups (Figure 8). Correlation analysis demonstrated that Se, miR-146a and TLR2 were positive associated (Figure 8B).

### Effect of Se and miR-146a inhibitor on *S. aureus* infected primary mammary epithelial cells

**Figure 9:**
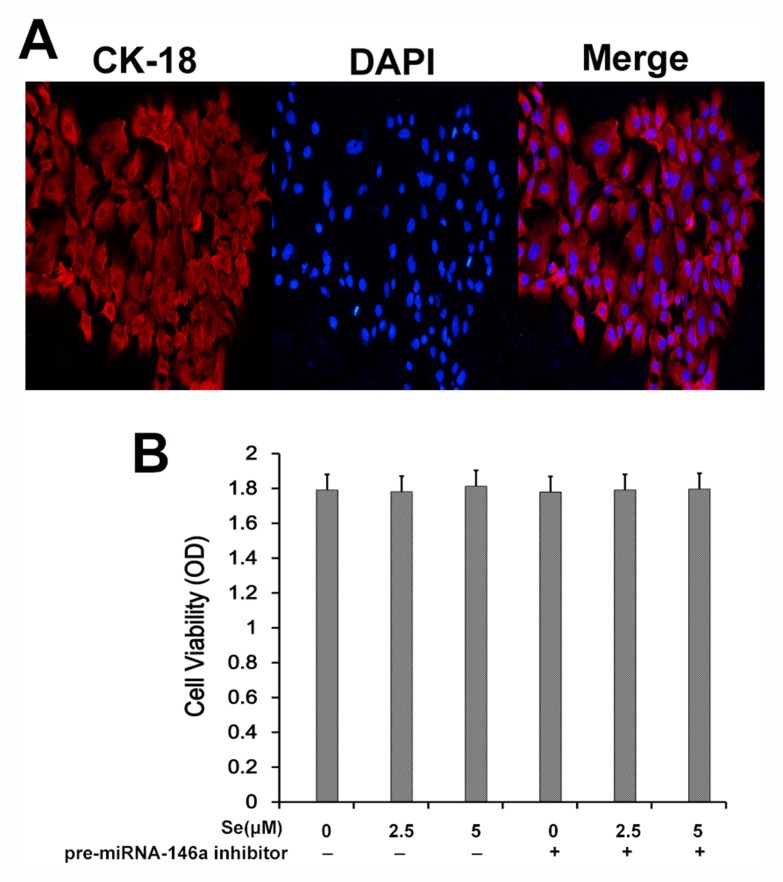
Effect of Se on *S. aureus*- infected primary mammary epithelial cells **(A)** Representative immunofluorescence image showing keratin 18 staining in primary mammary epithelial cells. **(B)** MTT assay showing cell viability in primary mammary epithelial cells treated with 0, 2.5 and 5 μM with or without miR-146 inhibitor.

Next, we determined effects of selenium and miR-146a inhibitor on *in vitro* cultured primary mammary epithelial cells. Immunofluorescence staining with keratin 18 showed that primary mammary epithelial cells isolated from mice were normal and of high purity (Figure 9A). MTT assay demonstrated that treatment with Se and miR-146a inhibitor did not affect viability of the primary mammary epithelial cells (Figure 9B).

### Se inhibits the inflammatory response in *S. aureus*-infected mammary epithelial cells by upregulating miR-146a

**Figure 10:**
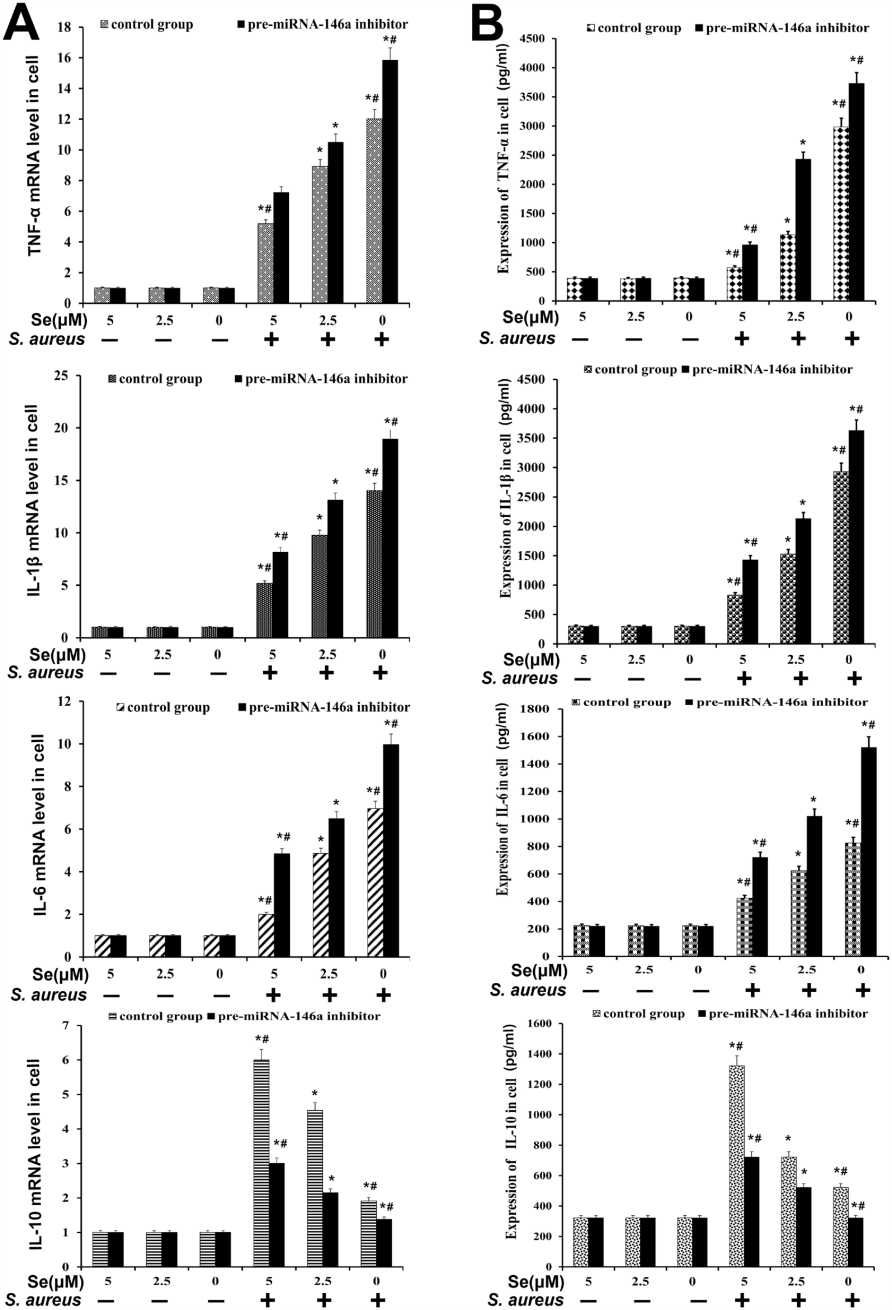
Effect of Se on inflammatory response in *S. aureus*- infected primary mammary epithelial cells **(A)** qRT-PCR and **(B)** ELISA analysis of TNF-α, IL-β, IL-6 and IL-10 mRNA and protein levels in *S. aureus* infected or uninfected primary mammary epithelial cells. Note: ^*^ denotes P<0.05 compared to respective control groups; ^#^ denotes P<0.05 compared to SNG group.

We determined the effects of Se and miR-146a on inflammatory response in *S. aureus* infected primary mammary epithelial cells. The cells were divided into 4 treatment groups: (1) Control group (no miR-146a inhibitor and uninfected); (2) miR-146a inhibitor without *S. aureus* infection; (3) *S. aureus-*infected group without miR-146a inhibitor and (4) miR-146a inhibitor with *S. aureus* infection. ELISA and qRT-PCR analysis showed high TNF-α, IL-1β, IL-6 and low IL-10 levels in the infected groups than uninfected groups (Figure 10). Se treatment decreased expression of pro-inflammatory cytokines (TNF-α, IL-1β and IL-6) in a concentration dependent manner (Figure 10). Moreover, miR-146 inhibition increased TNF-α, IL-1β, IL-6 and decreased IL-10 (Figure 10). These data demonstrated that both Se and miR-146a inhibited the inflammatory response in *S. aureus* infected mammary epithelial cells.

### Se inhibits TLR2 and TLR6 expression in *S. aureus*-infected mammary epithelial cells via miR-146a

**Figure 11:**
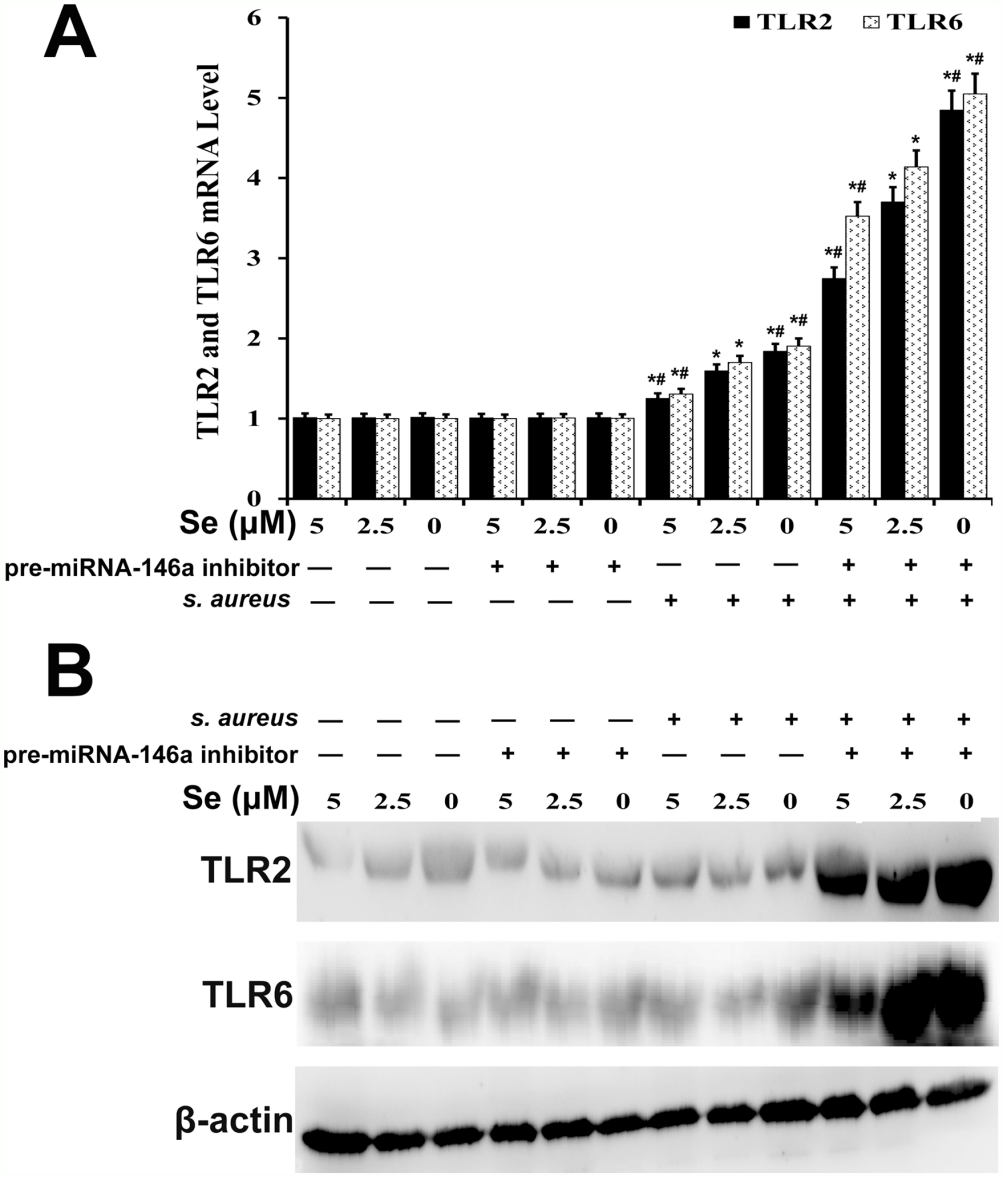
Effect of Se on TLR2 and TLR6 expression in *S. aureus*-infected primary mammary epithelial cells **(A)** qRT-PCR and **(B)** western blot analysis of TLR2 and TLR6 mRNA and protein levels, respectively in *S. aureus* infected or uninfected primary mammary epithelial cells. Note: ^*^ denotes P<0.05 compared to respective control groups; ^#^ denotes P<0.05 compared to SNG group.

We analyzed the effect of miRNA-146a on TLR2 and TLR6 mRNA and protein expression in *S. aureus-*infected mammary epithelial cells by qRT-PCR and western blot, respectively. We observed that TLR2 and TLR6 expression was higher in *S. aureus-*infected cells than in uninfected cells (Figure 11). Se decreased TLR2 and TLR6 expression in *S. aureus-*infected cells in a concentration dependent manner (Figure 11). Moreover, miR-146a inhibition diminished the effect of Se on TLR2 and TLR6 expression (Figure 11). This suggested that both miR-146a and Se decreased TLR2 and TLR6 levels in *S. aureus-*infected mammary epithelial cells.

### Se inhibits NF-κB and MAPK signaling in *S. aureus*-infected mammary epithelial cells via miR-146a

**Figure 12:**
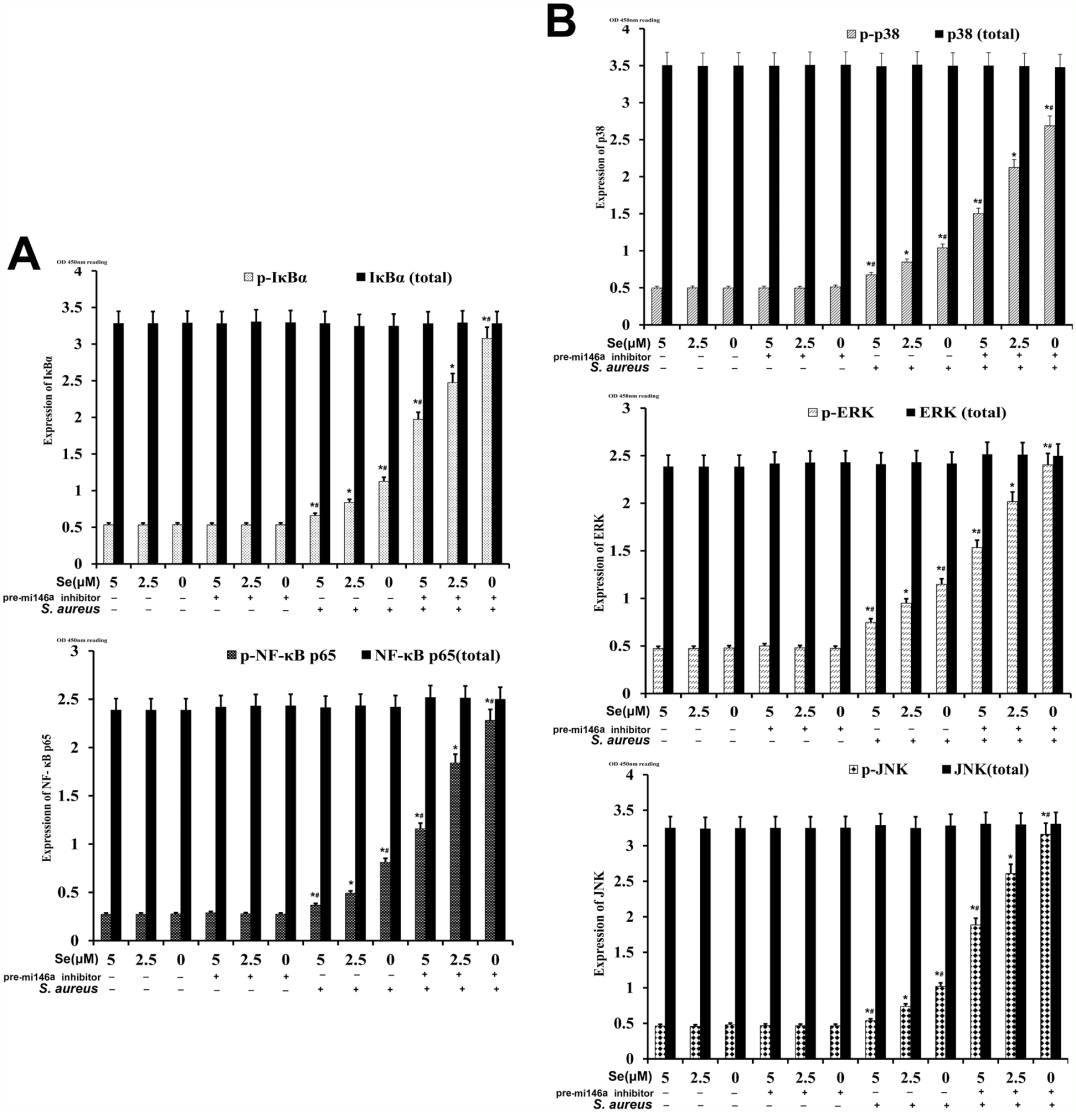
Effect of Se on NF-κB and MAPKs signaling in *S. aureus*-infected primary mammary epithelial cells ELISA analysis of total and phosphorylated **(A)** NF-κB p65 and IκB-α as well as **(B)** p38, ERK and JNK protein levels in *S. aureus* infected or uninfected primary mammary epithelial cells. Note: ^*^ denotes P<0.05 compared to respective control groups; ^#^ denotes P<0.05 compared to SNG group.

We further investigated the effect of miRNA-146a on NF-κB and MAPK signaling in mammary epithelial cells by analyzing total and phosphorylated of p65, IκBα, p38, JNK and ERK proteins by ELISA. We observed that the levels of p65, IκBα, p38, JNK and ERK proteins were similar in both *S-aureus*-infected and uninfected groups (Figure 12). But, the *S. aureus*-infected groups showed increased expression of phosphorylated p65, IκBα, p38, JNK and ERK than uninfected cells (Figure 12). Se treatment decreased phosphorylated forms of p65, IκBα, p38, JNK and ERK in a concentration dependent manner (Figure 12). Moreover, miR-146a inhibition decreased the inhibitory effect of Se on NF-κB and MAPK signaling (Figure 12). This demonstrated that Se decreased NF-κB and MAPK signaling in *S. aureus*-infected mammary epithelial cells via miR-146a.

## DISCUSSION

Many studies have demonstrated that Se reduces inflammation by decreasing the production of inflammatory cytokines [17, 18], regulating the immunoreaction [19, 20]. In this study, we demonstrated that Se inhibited inflammatory response to *S. aureus* infection in the mouse mastitis model and the mammary epithelial cells in a dose-dependent manner. Histopathological analysis showed that *S. aureus* infection resulted in mammary tissue damage, disruption of acinar structural organization, inflammatory cell infiltration and tissue cell necrosis. Se treatment decreased the pathology in a dose-dependent manner by significantly decreasing inflammation. The inflammatory response is regulated by the production of pro-inflammatory cytokines like TNF-α, IL-1β and IL-6, whereas the anti-inflammatory response is controlled by cytokines like IL-10 [17]. The immune response is regulated by the binding of multiple cytokines to their corresponding surface or intracellular receptors [21]. We analyzed TNF-α, IL-1β, IL-6 and IL-10 levels in mouse mastitis model and the mammary epithelial cells by qRT-PCR and ELISA. These cytokines play a major role in the regulation of inflammation caused by *S. aureus* [4–6]*.* We demonstrated that *S. aureus* infection induced TNF-α, IL-1β and IL-6 and decreased IL-10 levels. But, Se treatment decreased TNF-α, IL-1β and IL-6 and increased IL-10 levels in a dose dependent manner.

TLRs transduce inflammatory signals to activate NF-κB and MAPK signaling pathways, leading to the production of inflammatory cytokines [22]. TLR2 and TLR6 play a critical role during *S. aureus* infection [7, 8]. We demonstrated that *S. aureus* infection induced TLR2 and TLR6 in both *in vivo* and *in vitro* models. Se decreased TLR2 and TLR4 levels in a dose dependent manner in *S. aureus*-infected mammary tissues and cells. Many miRNAs play a critical role in regulating inflammation [23]. Moreover, Se modulates expression of some miRNAs [24]. We demonstrated that miR-146a expression correlated with Se treatment. In a previous study, it was postulated that Se regulated inflammation via miR-146a [25]. Our data showed that miR-146a inhibitor increased TLR2, TLR6, NF-κB and MAPK signaling pathways in *S. aureus*-infected Se treated primary epithelial cells. Correlation analysis demonstrated that both selenium and miR-146a co-coordinately regulate the anti-inflammatory response in *S. aureus* infected mouse mastitis and mammary epithelial cells. Therefore, we postulate that Se decreases inflammatory response in the mouse mastitis model by increasing miR-146a expression. Thus, our study demonstrates the therapeutic potential of Se supplementation to reduce and prevent *S. aureus* mastitis.

## MATERIALS AND METHODS

### Reagents

Se-(methyl) selenocysteine hydrochloride (MSC; purity≥95%, relative molecular mass=218.54) was purchased from Sigma-Aldrich Co. Ltd. (Shanghai, China). DMEM, TRIzol and fetal bovine serum (FBS) were obtained from Invitrogen. Penicillin and *S. aureus* were obtained from Sigma Co. (St. Louis, MO, USA). Mouse TNF-α, IL-1β and IL-6 enzyme-linked immunosorbent assay (ELISA) kits were obtained from Biolegend (San Diego, CA, USA). Rabbit mAb, IκBα, p65, p38, ERK, and JNK, and mouse monoclonal antibodies p-IκBα, p-p65, p-p38, p-ERK, and p-JNK were purchased from Cell Signaling Technology Inc (Beverly, MA, USA). β-actin and horseradish peroxidase conjugated goat anti-rabbit and goat anti-mouse antibodies were provided by Tianjin Sungene Biotech Co., Ltd. (Tianjin, China). The Nuclear and Cytoplasmic Protein Extraction Kit was provided by Beyotime Institute of Biotechnology (Jiangsu, China). All other chemicals were of reagent grade.

### Animals and experimental groups

Sixty male Balb/c mice (6 weeks old, ∼25g) were purchased from Animal Experiment Center, Huazhong Agricultural University (China). All experimental procedures were approved by the Institutional Animal Care and Use Committee of the Huazhong Agriculture University (20170319). The mice had free access to food and drinking water and were housed in micro-isolator cages under ambient conditions (temperature, 24±1°C; relative humidity, 40–80 %). Mice were allowed to acclimatize for 4–6 days before experimentation. The mice were randomly divided into three groups. Each group was fed a diet containing either a low (0.037 mg/kg), normal (0.15 mg/kg) or high (1.5 mg/kg) Se concentration for 90 days. The water provided to each group was without Se, namely, low level group (LG; 0.037 mg/kg Se), middle level group (MG; 0.150 mg/kg Se) and high level group (HG; 1.5 mg/kg Se). The mice were fed Se-supplement diet and drinking water for 60 days. Then, five of the ten mice in each group of 10 mice were injected with 100μl *S. aureus* suspension into the mammary duct with microsyringe for 48h. Finally, the experimental mice were sacrificed and their mammary tissues were removed and frozen at 80°C for further experiments.

### Histopathological evaluation of mammary tissue sections

A small portion of mammary tissue from each mouse was fixed in 10% formalin for histopathological analysis. The mammary tissues were dehydrated through graded alcohol (30-100% ethanol), embedded in paraffin. The paraffin-embedded specimens were serially sectioned. Some sections were stained with hematoxylin and eosin and the pathological changes were documented under an optical microscope. The other sections were analyzed for apoptosis by TUNEL assay. The paraffin-embedded tissues were dewaxed and rehydrated with decreasing percentages of ethanol (100-30%). Then, the tissue sections were incubated with, proteinase K, fixed with paraformaldehyde solution and permeabilized in sodium citrate solution. Then, they were labeled with the sections were labeled with the TUNEL reaction mixture use (FITC-conjugated anti-BrdU) and analyzed with a fluorescence microscope with an excitation/emission filter set at 530/630 nm.

### MPO analysis

We analyzed MPO, a neutrophil activation marker using the kit from Nanjing Jiancheng Bioengineering Institute, Nanjing, China. Briefly, we homogenized the mammary tissues from all groups of mice. The standards and the samples were added into the 96 well plate followed by incubation the anti-MPO antibody and the color reagent for 1 h. The supernatant was centrifuged and the MPO antibody was coated with 1/100 (v/v). Then, the OD of the samples were analyzed was measured in a spectrophotometer at 460 nm. All operations follow the manufacturer’s instructions (Nanjing Jiancheng Bioengineering Institute, Nanjing, China).

### Analysis of Se concentration in mouse mammary tissue

The Se concentration in the mammary tissues was detected by fluorescence spectrophotometry. It was measured following the method described by YAO et al [26]. The mammary tissue samples were extracted with Nitric acid and Perchloric acid (9:1 ratio) at 120°C for 2 h. The homogenate was evaporated at 160°C with constant stirring. After cooling, the powder was extracted with hydrochloric acid at 100°C to reduce hexavalent selenium to tetravalent Se. The solution was transferred to a small tube and analyzed in a fluorescence spectrophotometer to measure the selenium concentration. A standard curve was generated for various selenium concentrations and the Se content in the mammary samples was determined from the standard curve.

### Cell culture and treatments

The mouse mammary epithelial cells were isolated from all groups of mice. They were divided into three groups, which were grown in DMEM (Hyclone) medium supplemented with 10% fetal bovine serum (FBS) with 0, 2.5 or 10 μM Se at 37 °C and 5% CO2. After 24 hours, fresh medium was added to the cells and were divided into control (saline-treated) and *S. aureus* infected groups.

### MTT cell viability assay

MTT assay was used to determine the cytotoxic effects of Se on mammary epithelial cells. Mammary cells were assessed by MTT assay. Mammary epithelial cells (2 x 10^5^/ml) were seeded in 96-well plates at 37°C and 5% CO2 for 1h. After addition of MTT for 3 h, the cells were extracted with DMSO and analyzed at 570 nm in a microplate reader.

### Immunofluorescence labeling with keratin 18

For immunofluorescence labeling, mammary epithelial cells were labeled with fluorescently conjugated anti-keratin 18 antibodies (1/100 V/v, Invitrogen Inc, USA). Then, the cells were fixed with 3% paraformaldehyde for 10 minutes, and analyzed by laser confocal microscopy.

### MiRNA-146a inhibition

Mammary epithelial cells treated with 0, 2.5 and 5 μM Se from uninfected control mice groups. The cells were transfected with pre-miRNA-146a inhibitor to inhibit miR-146a expression, pretreatment by the transfection reagent lipo 2000 (Cell Signaling Technology Inc, Beverly, USA)). After 24 h, control and miR-146a inhibited cells were analyzed.

### ELISA

Mammary tissue samples were homogenized with phosphate-buffered saline (1/10, v/v) and centrifuged at 12000 rpm for 10 min. The supernatant was stored at -20 °C prior to the measurements. The inflammatory cytokines, TNF-α, IL-1β, IL-6 and IL-10 and NF-κB and MAPK pathway proteins, p65, IKKα, p38, ERK and JNK were analyzed by the corresponding ELISA kits (BioLegend, Inc., San Diego, CA, USA) according to manufacturer’s instructions. The experiments were performed thrice.

### Quantitative real time PCR

Total RNA was isolated from mammary tissue samples with TRIzol (Invitrogen, China) according to manufacturer’s instructions and the quantity and purity was determined in a UV spectrophotometer at 260/280 nm. RNA samples were reverse transcribed to complementary DNA (cDNA) by the Revert Aid First Strand cDNA Synthesis Kit (Invitrogen, Inc., CA, USA.). Specific primers were designed for TNF-α, IL-1β, IL-6 and IL-10 by Primer Premier Software (Premier Biosoft International, Paolo Alto, CA, USA) in Table 1. Real time PCR was performed in a 7500 Fast Real-Time PCR System (Applied Biosystems) with SYBR green Plus Kit (Roche). The PCR conditions included 1 cycle of 95 °C for 10 min followed by 40 cycles of 95°C for 15 s and 60°C for 60 s and the final cycle of 60 °C for 30 s. The experiment was repeated thrice. The relative expression of the experimental gene transcripts was expressed with GAPDH as the reference as 2^-ΔΔCt^.

**Table 1:** Oligonucleotide primers used for qPCR

Name	Primer sequence	product size (bp)
TNF-α	Sense: 5’- TGTGCCAGGGGACCATCTTCACG-3’	189
	Anti-sense: 5’- AAGCGAGGGGGGAGTCAGAGTT-3’	
IL-1β	Sense: 5’-GCCTCCAGGGGTCATCCTCAGTTCTA-3’	230
	Anti-sense: 5’- GGTTGTCAGGGAGGGAGCCCCCTTG-3’	
IL-6	Sense: 5’-ACCTGTGTGGGACTTTCCCGTGG-3’	213
	Anti-sense: 5’- TCATCAGGGGCCTGTAGTG-3’	
IL-10	Sense: 5’-AGCAATGGTTGTGCAATTCTGA-3’	197
	Anti-sense: 5’- ACTGGCGGACTTGGGATTGTCTTTCT-3’	
GAPDH	Sense: 5’-TAAAGCTCAGTAGGGAACAGTCGG-3’	203
	Anti-sense: 5’-TGCAATCGGCATCCATGGGAGAAAC-3’	

### Western blotting

Protein lysates were prepared from mammary tissues and quantified with a BCA Protein Assay Kit (Thermo, Rockford, USA). Equal amounts of protein lysates were separated by 12% and 10% SDS-PAGE at 100V for 2h and transferred onto PVDF membranes at 100V for 1 h. The blots were blocked with 5% skimmed milk in 1X TBST for 2 h. Then, the membranes were incubated with primary antibodies (1°C1000 dilution in 1X TBST) at 4°C for 12 h. After washing the membranes twice with TBST and once with TBS, the blots were incubated with HRP conjugated secondary antibodies (1°C5000 dilution in 1X TBST) for 1 h. After washing the membranes thrice, the blots were developed with ECL (GE, USA.) plus Image Quant LAS 4000 mini (GE, USA). The relative amounts of the proteins were measured with the NIH image quantification system β-actin as reference control.

### Statistical analysis

Statistical analysis was performed with the SPSS software (ver. 17 for Windows; SPSS Inc., Chicago, IL, USA). The differences between groups were determined by a one-way ANOVA and a p < 0.05 was considered statistically significant. To compare multiple data groups, Tukey–Kramer method was used. The data are expressed as mean±SD.
